# Inbreeding depression of sperm traits in the zebra finch *Taeniopygia guttata*

**DOI:** 10.1002/ece3.1868

**Published:** 2015-12-29

**Authors:** Pavlína Opatová, Malika Ihle, Jana Albrechtová, Oldřich Tomášek, Bart Kempenaers, Wolfgang Forstmeier, Tomáš Albrecht

**Affiliations:** ^1^ Institute of Vertebrate BiologyAcademy of Sciences of the Czech Republicv.v.i.Květná 8CZ‐603 65BrnoCzech Republic; ^2^ Department of Botany and ZoologyFaculty of ScienceMasaryk UniversityKotlářská 267/2CZ‐61137BrnoCzech Republic; ^3^ Department of Behavioural Ecology and Evolutionary GeneticsMax Planck Institute for OrnithologyEberhard‐Gwinner‐Strasse 782319SeewiesenGermany; ^4^ Charles University in PragueFaculty of SciencesDepartment of ZoologyViničná 7CZ‐12844PragueCzech Republic

**Keywords:** Gamete performance, genetic quality, sperm abnormality, sperm morphology, sperm velocity

## Abstract

Inbreeding depression, or the reduction in fitness due to mating between close relatives, is a key issue in biology today. Inbreeding negatively affects many fitness‐related traits, including survival and reproductive success. Despite this, very few studies have quantified the effects of inbreeding on vertebrate gamete traits under controlled breeding conditions using a full‐sib mating approach. Here, we provide comprehensive evidence for the negative effect of inbreeding on sperm traits in a bird, the zebra finch *Taeniopygia guttata*. We compared sperm characteristics of both inbred (pedigree *F* = 0.25) and outbred (pedigree *F* = 0) individuals from two captive populations, one domesticated and one recently wild‐derived, raised under standardized conditions. As normal spermatozoa morphology did not differ consistently between inbred and outbred individuals, our study confirms the hypothesis that sperm morphology is not particularly susceptible to inbreeding depression. Inbreeding did, however, lead to significantly lower sperm motility and a substantially higher percentage of abnormal spermatozoa in ejaculate. These results were consistent across both study populations, confirming the generality and reliability of our findings.

## Introduction

Inbreeding, or mating between close relatives, is known to have a detrimental effect on the phenotypes and performance traits of offspring (Keller and Waller 2002). Under certain circumstances, inbreeding depression can be strong enough to drive populations to extinction (Saccheri et al. 1998; Frankham 2005). Hence, knowledge of the extent to which traits are susceptible to inbreeding depression has wide implications for evolutionary (Charlesworth and Charlesworth 1987; Lynch and Walsh 1998) and conservation biology (Crnokrak and Roff 1999; Bijlsma et al. 2000; Leberg and Firmin 2008; Knief et al. 2015) and agricultural sciences (Sewalem et al. 1999; König et al. 2010; Makina et al. 2014).

Inbreeding depression is thought to arise primarily from deleterious recessive mutations whose detrimental effects are only revealed once they become homozygous (identical by descent) in inbred individuals (Falconer and Mackay 1996; Lynch and Walsh 1998; Charlesworth and Willis 2009). Traits under directional selection, such as fitness, typically show a decline in performance with decreasing condition (Rowe and Houle 1996). High performance depends on the proper functioning of numerous genes, any of which can be knocked out by deleterious mutations. Furthermore, mutations that may improve performance are much less likely to arise. In contrast, while traits under stabilizing selection, such as morphological traits, may be subject to perturbation from mutations, the direction of change is often random, leading to an increase in variance but not necessarily to a change in mean trait value. As a result, traits closely associated with fitness and under directional selection and traits that are condition‐dependent are predicted to show stronger inbreeding depression than those weakly associated with fitness and/or under stabilizing selection (Falconer and Mackay 1996; Ala‐Honkola et al. 2013).

There is ample evidence that inbreeding affects a range of fitness‐related traits in normally outbreeding organisms, including birth weight, developmental time, survival, resistance to disease and fecundity (Keller and Waller 2002; Wright et al. 2008). Inbreeding is also associated with reduced expression of precopulatory sexual signals, such as male sexual ornamentation and courtship rate (Bolund et al. 2010). Male fitness also depends on postcopulatory processes (Birkhead 2010) and both sperm quality (sperm velocity or morphometry) and sperm quantity have been shown to affect male reproductive performance in a range of animal taxa (Parker 1998; Snook 2005; Michalczyk et al. 2010), including birds (Denk et al. 2005; Pizzari et al. 2008; Bennison et al. 2015).

Although the sperm cell itself is haploid, with limited gene expression, gametogenesis is under diploid genetic control (Nayernia et al. 1996) and could be prone to the adverse effects of inbreeding such as reduced sperm quality. Previous studies have shown that both sperm concentration and morphology are adversely affected by inbreeding in a range of animal taxa (Losdat et al. 2014). Several studies also report an inbreeding effect on measures of sperm functioning, such as the percentage of motile sperm and/or the proportion of spermatozoa with progressive motility (Wildt et al. 1982; Gomendio et al. 2000; van Eldik et al. 2006; Maximini et al. 2011). Information on how inbreeding affects sperm swimming speed (velocity), however, is largely missing (but see Gasparini et al. 2013). In addition to its effect on sperm morphology or function, failures in spermatogenesis may lead to azoospermia and/or the occurrence of abnormal spermatozoa in the ejaculate, thus reducing the number of sperm available to fertilize ova (Gage et al. 2006). Similarly, expression of developmental errors during spermatogenesis in inbred individuals could explain increased within‐ejaculate variability in sperm length (Fitzpatrick and Evans 2009; Michalczyk et al. 2010).

A recent meta‐analysis evaluating the effects of inbreeding on gamete quality included studies on 16 vertebrate species, with mammals and fish clearly prevailing (Losdat et al. 2014). Birds in particular remain a neglected taxonomic group in such studies, despite being a model for multiple mating and sperm competition studies (Griffith et al. 2002; Immler et al. 2011). In contrast with studies on invertebrates and plants, most such studies have not used controlled breeding designs (e.g., full‐sib mating) to produce inbred and outbred individuals raised under standard conditions (but see Zajitschek et al. 2009; Mehlis et al. 2012; Gasparini et al. 2013). The effects of inbreeding are thus typically inferred from incidental observations rather than from balanced experimental data (typically relating genetic marker‐derived inbreeding coefficients with the sperm trait of interest; Slate and Pemberton 2006).

The aim of our study was to generate robust data allowing a comprehensive evaluation of the effects of inbreeding on key sperm traits in a songbird, the zebra finch *Taeniopygia guttata*. Zebra finches are known to suffer from inbreeding depression in several fitness‐related traits (Bolund et al. 2010). Following the suggestion that comparisons between inbred and noninbred individuals should be appropriately controlled or conducted under standardized conditions (Gage et al. 2006; Slate and Pemberton 2006), we used one generation of full‐sib mating to produce inbred individuals and compared them with outbred individuals raised and kept under the same conditions. We used two independent zebra finch populations, one domesticated and the other recently wild‐derived, in order to obtain two independent estimates of the magnitude of inbreeding depression on sperm traits and to reduce the risk of false positive results (see Fanelli 2011). We focussed on two types of sperm trait: (1) traits for which evidence of the effects of inbreeding already exist in other animal species (e.g., percentage of abnormal sperm cells in semen, length of “normal” sperm, within‐male variation in sperm length; reviewed in Losdat et al. 2014); and (2) traits for which evidence of an association with male fertilization capability already exists in zebra finches (e.g., sperm length; Bennison et al. 2015) or other bird species (e.g., sperm swimming speed; Denk et al. 2005; Pizzari et al. 2008).

## Material and Methods

### Study populations

We studied two independent captive populations, one domesticated and one recently wild‐derived, held at the Max Planck Institute for Ornithology in Seewiesen, Germany (birds originated from populations #4 and #18 described in Forstmeier et al. 2007). Inbred individuals (via full‐sib mating, *F* = 0.25) and outbred controls (*F* = 0, according to a 3–5 generations pedigree) were produced from both populations. Note, however, that captive populations typically show a baseline level of inbreeding that is not captured by the pedigree information due to co‐ancestry of pedigree founders. Based on molecular data, this baseline appears to lie somewhere between *F* = 0.05 to *F* = 0.1 for our domesticated population (Knief et al. 2015), and probably slightly lower for the recently wild‐derived population (Forstmeier et al. 2007). Assuming a conservative baseline of *F* = 0.1, therefore, we would be comparing inbreds of about *F* = 0.325 (0.1 + 0.9*0.25 = 0.325) to outbreds of *F* = 0.1. This would result in an underestimation of effect size by a factor of 0.9 as we assume a difference in *F* of 0.25, whereas it may actually be only 0.225. Selective mortality of those individuals with the highest proportion of this genome being identical by descent (due to Mendelian sampling noise) would provide a further source of underestimation for inbreeding effect.

In the domesticated population, we took sperm samples from 16 inbred males (age range 630–1375 days; mean 1132 days) originating from full‐sib mating of 11 different families. For comparison, we sampled 20 age‐matched (633–1340 days; mean 1176 days) outbred males (from 13 families) that had been raised and held under the same conditions. Most of these birds (11 inbreds, 15 outbreds) originated from uncontrolled mating in communal aviaries where birds could choose between inbreeding and outbreeding with a 50% chance probability (a potential source of bias in the data; Bolund et al. 2010). All males were sampled for sperm just once.

In the recently wild‐derived population, we employed a more controlled design whereby we randomly allocated parents to inbreed (12 pairs) or outbreed (24 pairs) in separate aviaries (see Ihle et al. 2013). We then sampled sperm from 23 inbred male offspring (all those alive in April 2012) from seven different families (initially 196–367 days old, mean 286 days) and 20 outbred males (alive and matched for housing conditions) from 14 different families (184–366 days, mean 318 days). Each male was sampled three times: in April 2012, August 2012 and April 2013. Sample sizes differed between sampling periods, either because individuals died (*n* = 3) or because we were unable to maintain the same standardized housing conditions (*n* = 7). Throughout the study, and for both populations, we ensured that inbreds and outbreds were always matched for recent breeding history and opportunity to copulate.

Across the two populations, we were unable to obtain a sperm sample during 12 of 148 attempts (four inbred and three outbred males never yielded any sperm). In addition, 10 sampling attempts led to incomplete data (e.g., morphology but no velocity measurement).

### Analysis of sperm traits

With regard to bird inbreeding status, all handling and measurement of sperm characteristics was undertaken blind. In each case, fresh semen was collected by massaging the cloacal protuberance. The sperm (ca. 0.5–3 *μ*l) was diluted within 20 s of collection in preheated (40°C) Dulbecco's Modified Eagle's Medium (Advanced DMEM, Invitrogen). A sample was then pipetted onto a standard 20 *μ*m two‐chamber count slide (Leja, The Netherlands) for analysis of velocity (see below). A further aliquot of fresh semen was fixed in 250 *μ*L of ~5% formalin for analysis of morphology and proportion of abnormal sperm.

Sperm velocity was recorded immediately after dilution of the freshly obtained sperm sample in DMEM. Each sperm sample was recorded for approximately 45 s in eight different Leja slide fields using a 100× magnification CX41 Olympus microscope (Olympus, Tokyo, Japan) fitted with a heating table (kept at a constant temperature of 40°C), phase contrast and a UI‐1540‐C Olympus digital camera (Olympus, Japan). Each recording field was later analysed using the CEROS computer‐assisted sperm analysis system (Hamilton Thorne Inc., Beverly, Massachusetts, USA). Each object tracked was visually inspected and nonsperm objects were manually excluded from the analysis. Spermatozoa with a straight line velocity of <20.5 *μ*m s^−1^ were considered static and excluded from motility analysis. The choice to remove immotile sperm from velocity measurement was made a priori as it allows for the study of sperm velocity independent of sperm abnormalities. As the in vitro medium (DMEM) does not contain spermatozoa attractants (see also Laskemoen et al. 2010), only curvilinear velocity (VCL) was used for statistical analysis of each male's sperm.

Sperm morphology was measured using Olympus QuickPHOTO Industrial 2.3 imaging software. For each sample, we measured the length of the head, mid‐piece, and tail and calculated total sperm length as the sum of the three components on ten intact (normal) spermatozoa (see also Laskemoen et al. 2010). The proportion of morphologically normal and abnormal spermatozoa in sperm samples was assessed under a 400x magnification BX51 Olympus light microscope (Olympus, Japan), with 100 sperm cells analysed per sample for each bird. Each sperm not showing the typical helical songbird head‐shape was considered abnormal, as were the few sperm cells showing tail deformities (two‐tailed spermatozoa with one head and one mid‐piece). All scoring was done by the same person (PO) in order to reduce observer error. A subset of 16 slides (eight inbred and eight outbred males) were scored a second time, again inspecting a random subset of 100 spermatozoa on each slide. This yielded a scoring repeatability of 95% (*R *=* *0.95, SE = 0.025, *P* < 0.001; rptR package, REML method; Nakagawa and Schielzeth 2010).

### Statistical analysis

For all morphological traits we calculated the mean trait value for each male within each sampling event. Coefficients of variance (CV) were calculated by dividing within‐male standard deviation by the average trait value multiplied by 100. Log‐transformation of CVs and of abnormal sperm counts ensured normality of all dependent variables. In the wild‐derived population, we assessed sperm trait repeatability across the three sampling sessions using the rptR package (Nakagawa and Schielzeth 2010) in R 3.0.2 (R Development Core Team 2013), without accounting for effects of inbreeding or sampling session. To quantify the effect of inbreeding, we used generalized linear (mixed‐effect) models (using the lmer function; Bates et al. 2013) with male and family identity as random effects and sampling session (three levels, wild‐derived population only) and inbreeding (two levels) as fixed effects. We also ran identical models on the entire data set, accounting for population effects by defining a fourth level in the predictor “sampling session.” We calculated effect size (Cohen's *d*) by dividing the parameter estimate for the effect of inbreeding by the average phenotypic standard deviation calculated within groups and within sampling sessions. Note that we chose not to test for population differences or effects of age as these are completely or strongly linked with possible effects of sampling session. We refer to this type of variation as a session effect in order to avoid suggesting that population differences or age effects could be estimated reliably.

## Results

Nine of ten sperm traits (all except CV of head length) measured repeatedly across a one‐year period in the recently wild‐derived population showed significant long‐term repeatability, ranging from *r *=* *0.22 (sperm velocity) to *r *≈* *0.8–0.9 (sperm size; see Table 1).

**Table 1:** Repeatability of sperm traits in wild‐derived male zebra finches. *P*‐values are from permutation tests. CV, coefficient of variance; SE, standard error

Sperm trait	*n* measures	*n* males	Repeatability	SE	*P*
Total sperm length	104	40	0.83	0.046	<0.001
Head length	104	40	0.77	0.060	<0.001
Midpiece length	104	40	0.90	0.030	<0.001
Tail length	104	40	0.93	0.022	<0.001
Log CV total length	104	40	0.29	0.107	0.008
Log CV head length	104	40	0.12	0.099	n.s.
Log CV midpiece length	104	40	0.47	0.107	<0.001
Log CV tail length	104	40	0.22	0.109	0.018
Log (% abnormal)	103	40	0.34	0.119	<0.001
Velocity [*μ*m/s]	97	38	0.22	0.117	0.031

**Figure 1:**
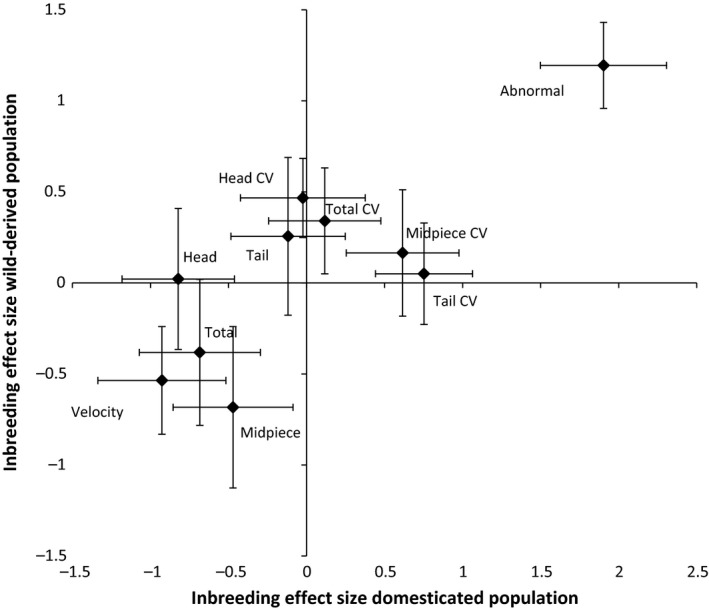
Comparison of inbreeding effect (Cohen's *d *± SE) on sperm phenotypic traits in a domesticated and wild‐derived zebra finch population.

Greatest inbreeding depression (average effect size *d* = 1.40; Table 2) was observed in the proportion of abnormal sperm, with inbred males averaging 22% and outbred males 8% (Table 3). Sperm velocity was also significantly reduced by inbreeding depression (average *d *=* *−0.74; Table 2 and Tables 4, 5, 6). In contrast, morphological traits (average *d *=* *−0.34; Table 2) and within‐male CV (average *d *=* *0.24; Table 2) differed only slightly between inbred and outbred males, the difference being nonsignificant. Some morphological traits appear to have been affected by inbreeding in one population but unaffected in the other, suggesting that the underlying effects were small or that the findings were not robust. Overall, inbreeding depression on sperm traits was similar across the two study populations (*r *=* *0.81, *n *=* *10 traits, *P *=* *0.005; Fig. 1), indicating that traits differ significantly in their sensitivity to inbreeding stress.

**Table 2:** Differences in sperm phenotypic traits between inbred and outbred male zebra finches in a domesticated and a wild‐derived population. Results are based on linear mixed effect models with male and family identity as random effects and sampling session (wild‐derived population only) and male inbreeding status as explanatory variables. Results of full models are provided in Tables S4–S6. CV, coefficient of variance. Values in bold highlight significant differences for 10 tests per population after Bonferroni correction

Sperm trait	Domesticated		Wild‐derived		Joint analysis	
	Cohen's *d*	*P*	Cohen's *d*	*P*	Cohen's *d*	*P*
Total sperm length	−0.68	0.078	−0.38	0.341	−0.55	0.069
Head length	−0.82	0.022	0.02	0.956	−0.41	0.164
Midpiece length	−0.47	0.220	−0.68	0.123	−0.49	0.084
Tail length	−0.12	0.746	0.26	0.555	0.08	0.784
Log CV total length	0.12	0.745	0.34	0.241	0.24	0.293
Log CV head length	−0.02	0.953	0.47	0.031	0.27	0.192
Log CV midpiece length	0.62	0.089	0.16	0.636	0.22	0.345
Log CV tail length	0.75	0.016	0.05	0.857	0.22	0.311
Log (% abnormal)	**1.90**	**<0.001**	**1.19**	**<0.001**	**1.40**	**<0.001**
Velocity [*μ*m/s]	−0.93	0.024	−0.54	0.070	−**0.74**	**0.002**

**Table 3:** Mean value and standard deviation (SD) of sperm traits in the domesticated and wild‐derived zebra finch populations. As measurements were taken over three different sessions for the wild‐derived population, SD was calculated within each session and an average taken. SD was then used for calculating effect size. Ranges (minimum‐maximum), indicated as sample sizes (*n*) vary slightly between sperm traits. CV, coefficient of variance. All values are in the original scale (without log‐transformation)

	Domesticated mean [SD]		Wild‐derived mean [SD]	
	Outbred	Inbred	Outbred	Inbred
*n* (males)	16–18	14	19	19–21
*n* (sperm samples)	16–18	14	52–53	45–51
Total sperm length [*μ*m]	65.87 [3.52]	62.86 [6.59]	67.24 [3.29]	68.11 [4.83]
Head length [*μ*m]	11.88 [0.75]	11.29 [0.80]	11.35 [0.51]	11.53 [0.66]
Midpiece length [*μ*m]	30.93 [3.97]	29.05 [5.48]	29.1 [4.54]	28.71 [4.44]
Tail length [*μ*m]	23.06 [6.29]	22.52 [7.64]	26.79 [6.82]	27.87 [5.93]
Total length CV	3.97 [1.52]	4.13 [1.80]	3.34 [1.17]	3.94 [1.71]
Head length CV	6.44 [1.53]	6.55 [2.35]	4.94 [1.40]	5.86 [2.50]
Midpiece length CV	9.67 [4.91]	11.35 [4.06]	11.79 [6.38]	11.89 [7.49]
Tail length CV	15.82 [7.59]	18.83 [6.00]	14.37 [5.66]	13.83 [4.73]
Proportion abnormal [%]	5.18 [3.26]	19.5 [11.71]	10.00 [9.95]	23.61 [15.01]
Velocity [*μ*m/s]	78.29 [15.4]	62.92 [17.3]	75.51 [16.29]	66.22 [12.14]

**Table 4:** Estimates from mixed effect models for sperm traits in a domesticated zebra finch population. The variance components for the random effect of family identity (var family ID) is shown compared with residual variance (var residual). Fixed effect parameter estimates, with standard error (SE), are given for the intercept for inbreeding effect. CV, coefficient of variance

Sperm trait	var family ID	var residual	Intercept [SE]	Inbreeding [SE]
Total sperm length	14.23	9.27	66.2 [1.31]	−3.46 [1.96]
Head length	0.041	0.535	11.9 [0.19]	−0.64 [0.28]
Midpiece length	4.50	18.35	31.1 [1.22]	−2.23 [1.82]
Tail length	4.37	42.95	23.4 [1.72]	−0.83 [2.55]
Log CV total length	0.0000	0.0294	0.56 [0.04]	0.02 [0.06]
Log CV head length	0.0118	0.0111	0.79 [0.04]	0.00 [0.06]
Log CV midpiece length	0.0011	0.0282	0.92 [0.04]	0.11 [0.06]
Log CV tail length	0.0000	0.0174	1.14 [0.03]	0.12 [0.05]
Log (% abnormal)	0.0289	0.0483	0.72 [0.07]	0.51 [0.11]
Velocity [*μ*m/s]	134.60	138.70	77.8 [4.58]	−15.1 [6.71]

**Table 5:** Estimates from mixed effect models for sperm traits in a wild‐derived zebra finch population. Variance components for the random effects of male identity (var male ID) and family identity (var family ID) are shown alongside residual variance (var residual). Fixed effect parameter estimates with standard error (SE) are given for the intercept, the difference between the second and third sessions and the first session and for inbreeding effect. CV, coefficient of variance

Sperm trait	var male ID	var family ID	var residual	Intercept [SE]	Session 2 [SE]	Session 3 [SE]	Inbreeding [SE]
Total sperm length	4.51	10.89	1.84	66.5 [1.08]	2.18 [0.33]	2.17 [0.34]	−1.55 [1.63]
Head length	0.212	0.107	0.073	11.3 [0.15]	−0.03 [0.07]	0.20 [0.07]	0.01 [0.23]
Midpiece length	7.80	15.90	1.55	28.7 [1.31]	0.95 [0.3]	1.32 [0.31]	−3.07 [1.99]
Tail length	15.91	29.89	2.45	26.5 [1.82]	1.26 [0.38]	0.63 [0.39]	1.63 [2.76]
Log CV total length	0.0036	0.0036	0.0194	0.50 [0.04]	0.01 [0.03]	0.03 [0.03]	0.05 [0.05]
Log CV head length	0.0020	0.0000	0.0184	0.66 [0.03]	0.04 [0.03]	−0.01 [0.03]	0.07 [0.03]
Log CV midpiece length	0.0150	0.0165	0.0278	1.05 [0.06]	−0.09 [0.04]	−0.03 [0.04]	0.04 [0.08]
Log CV tail length	0.0034	0.0029	0.0190	1.13 [0.03]	−0.01 [0.03]	0.00 [0.03]	0.01 [0.04]
Log (% abnormal)	0.0211	0.0000	0.0644	1.08 [0.06]	−0.18 [0.06]	−0.31 [0.06]	0.35 [0.07]
Velocity [*μ*m/s]	9.40	34.33	164.37	71.5 [3.14]	6.33 [3.11]	−1.39 [3.26]	−7.62 [4.20]

**Table 6:** Estimates from mixed effect models for sperm traits obtained from the joint analysis of the two zebra finch populations (domesticated and wild‐derived). The variance components for the random effects of male identity (var male ID) and family identity (var family ID) are shown alongside residual variance (var residual). Fixed effect parameter estimates with standard error (SE) are given for the intercept, the contrast of each of the three sessions of the wild‐derived population relative to the domesticated population, and for inbreeding effect. CV, coefficient of variance

Sperm trait	var male ID	var family ID	var residual	Intercept [SE]	Session 1 [SE]	Session 2 [SE]	Session 3 [SE]	Inbreeding [SE]
Total sperm length	5.42	13.82	1.86	65.5 [1.09]	1.31 [1.35]	3.49 [1.36]	3.47 [1.36]	−2.36 [1.30]
Head length	0.277	0.146	0.074	11.7 [0.16]	−0.26 [0.19]	−0.29 [0.19]	−0.06 [0.19]	−0.26 [0.19]
Midpiece length	11.05	9.28	1.55	31.0 [1.08]	−2.46 [1.31]	−1.52 [1.32]	−1.15 [1.32]	−2.23 [1.29]
Tail length	23.16	19.38	2.46	22.7 [1.55]	4.10 [1.89]	5.37 [1.89]	4.74 [1.90]	0.51 [1.86]
Log CV total length	0.0042	0.0031	0.0198	0.56 [0.03]	−0.06 [0.04]	−0.05 [0.04]	−0.02 [0.04]	0.04 [0.04]
Log CV head length	0.0013	0.0014	0.0186	0.77 [0.03]	−0.1 [0.04]	−0.05 [0.04]	−0.10 [0.04]	0.04 [0.03]
Log CV midpiece length	0.0153	0.0067	0.0265	0.96 [0.05]	0.08 [0.06]	−0.01 [0.06]	0.05 [0.06]	0.05 [0.05]
Log CV tail length	0.0037	0.0023	0.0190	1.19 [0.03]	−0.07 [0.04]	−0.08 [0.04]	−0.07 [0.04]	0.03 [0.03]
Log (% abnormal)	0.0200	0.0000	0.0633	0.77 [0.06]	0.28 [0.07]	0.09 [0.07]	−0.03 [0.07]	0.40 [0.06]
Velocity [*μ*m/s]	10.12	41.86	170.28	75.4 [3.32]	−2.68 [4.00]	3.64 [4.10]	−4.19 [4.22]	−10.9 [3.55]

After controlling for inbreeding, sperm velocity was not directly related to the proportion of abnormal sperm (*r *=* *−0.06, *n *=* *125, *P *=* *0.61; Table 7), suggesting that the effect of inbreeding on sperm velocity and sperm abnormalities occurs independently. Only when testing across the two groups were mean velocity and proportion of abnormal sperm correlated (*r *=* *−0.20, *n *=* *125, *P *=* *0.05; Table 7), with inbreeding affecting both traits (Table 2). Contrary to expectation (Bennison et al. 2015), sperm velocity was not correlated with sperm length (*r *=* *0.11, *n *=* *126, *P *=* *0.32; Table 7).

**Table 7:** Effect of other sperm traits on zebra finch sperm velocity, estimated from nine mixed‐effect models controlling for male and family identity, population (not significant), and session effect (not significant). Effect size estimates from models that control for inbreeding status as a fixed effect (always *P *<* *0.005) are shown under “*r* within,” and those from models that do not control for inbreeding are shown under “*r* across.” CV, coefficient of variance

Sperm trait	*n* measures	*n* males	*r* across	*P*	*r* within	*P*
Total sperm length	126	67	0.14	0.22	0.11	0.32
Head length	126	67	0.11	0.24	0.10	0.28
Midpiece length	126	67	0.16	0.10	0.12	0.21
Tail length	126	67	−0.06	0.57	−0.04	0.71
Log CV total length	126	67	−0.06	0.48	−0.05	0.56
Log CV head length	126	67	−0.05	0.54	−0.03	0.74
Log CV midpiece length	126	67	0.04	0.70	0.06	0.51
Log CV tail length	126	67	0.14	0.12	0.15	0.09
Log (% abnormal)	125	67	−0.20	0.05	−0.06	0.61

## Discussion

Our study, which was based on a controlled breeding design with offspring raised under standardized conditions, demonstrated a clear effect of inbreeding on the sperm characteristics of a songbird species, with inbred males having more abnormal spermatozoa and lower sperm velocity than outbred males kept under the same conditions. Furthermore, we showed that inbreeding depression affected sperm traits similarly in both domesticated and recently wild‐derived populations, supporting the general robustness of our findings (Fig. 1). The effect of inbreeding on the proportion of abnormal spermatozoa in ejaculate was approximately two‐times greater than that on sperm velocity. These two traits were not correlated in our populations; hence, inbreeding appears to affect semen quality in these two attributes independently. In contrast, we observed no significant effect of inbreeding on the morphology of normal‐looking sperm (potential effects were either weak or inconsistent between the two populations).

An ability to quantify strength of inbreeding depression could be useful for identifying traits that show condition‐dependent expression and are likely to be associated with fitness (Wright et al. 2008; Ala‐Honkola et al. 2013). Accordingly, one could conclude that proportion of abnormal spermatozoa and sperm velocity represent gamete traits associated with reproductive performance in zebra finch males. Indeed, both traits can significantly affect male fertilization ability and success. For example, a large proportion of abnormal sperm will reduce effective ejaculate size (Gage et al. 2006). Male fertilization success under sperm competition (but also in noncompetitive contexts) depends upon sperm number (Parker 1982), and hence on the proportion of normal sperm in the ejaculate (Malo et al. 2005; Pizzari et al. 2008). Abnormal sperm cells will have difficulty reaching the ovum due to compromised direction of movement (Saacke et al. 1994), lack of size compatibility with the female's sperm storage tubules (SSTs; Briskie et al. 1997) or an inability to penetrate the perivitelline membrane due to acrosomal dysfunction (Roldan et al. 1994).

High sperm velocity may also have a positive effect on sperm performance in both external and internal fertilizers (Snook 2005; Pizzari and Parker 2009). In birds, sperm velocity may be associated with both the ability to reach a female's SSTs and the ability to stay in the SSTs longer, which would provide a fertilization advantage to the male (Pizzari et al. 2008). Indeed, using artificial insemination in mallards *Anas platyrhynchos*, Denk et al. (2005) showed that, after experimentally controlling for variation in sperm number, sperm velocity was a key predictor of male fertilization success under sperm competition. This quality trait has tended to be neglected in studies focusing on the effects of inbreeding on sperm performance. The only other study that has compared sperm velocity in inbred and outbred individuals found no effect of inbreeding in guppies *Poecille reticulata* (Gasparini et al. 2013). A small number of correlative studies on other vertebrate species have used indirect proxies to estimate sperm velocity (such as the proportion of motile sperm in ejaculate), with the results showing inconsistent effects of inbreeding (Wildt et al. 1982; Gomendio et al. 2000; van Eldik et al. 2006). In birds, sperm velocity appears to be sensitive to high levels of oxidative stress (Helfenstein et al. 2010); here, we show that it may also be sensitive to genetic stress caused by inbreeding.

While tail length showed no sign of inbreeding depression (no change in mean trait value), head length, mid‐piece length and total sperm length all showed a (nonsignificant) trend toward lower values (mean effect size *d* = −0.48). However, given the problems inherent in testing multiple hypotheses, these trends should be treated with caution until further data becomes available, which will allow a final decision on whether these traits are weakly condition dependent or not. In line with a conservative interpretation of the results, no effect of inbreeding was reported on sperm length for fruit flies *Drosophila melanogaster* (Ala‐Honkola et al. 2013); the authors interpreting this result as supporting the concept of stabilizing selection on total sperm length in this species (Ala‐Honkola et al. 2013). In theory, traits under stabilizing selection should be less sensitive to inbreeding depression than those under directional selection (Falconer and Mackay 1996; Wright et al. 2008). Indeed, comparative studies across songbirds have indicated that within‐ and between‐male variance in sperm length is removed through postcopulatory stabilizing selection acting against extreme sperm length (Birkhead et al. 2005; Calhim et al. 2007; Immler et al. 2008; Kleven et al. 2008; Lifjeld et al. 2010).

Although our results suggest that sperm length itself is not prone to inbreeding depression in zebra finches, implying the absence of strong directional selection in this trait (Falconer and Mackay 1996), a recent study on the same species using artificial selection lines for sperm length found that longer sperm displayed a fertilization advantage over shorter sperm (Bennison et al. 2015). As the authors explain, however, the effect of sperm length on fertilization success could have been mediated through sperm velocity, as velocity was significantly higher in their long‐sperm selection line. Unlike sperm length, velocity did show signs of inbreeding depression in our study (see above). Interestingly, we found no association between sperm velocity and sperm length in zebra finches, despite considerable among‐male variation in average sperm length in our two populations (range 52–77 *μ*m, *n *=* *126). Hence, in principle, it should be possible to separate the effects of sperm length and sperm velocity on fertilization success in our populations.

One might expect that inbreeding would lead to an increase in trait variance, despite not having an effect on mean trait value. In our study, we found only a weak and nonsignificantly higher variance in sperm morphology traits within inbred males (mean effect size *d* = 0.24, Table 2) and only a slightly higher variance among inbred males (SD among inbred males was 27% higher than SD among outbred males; see Table 3). The weakness of effect may be attributable, in part, to the fact that we only measured normal‐looking sperm and excluded abnormal sperm, which was more frequent in inbred males. Increased within‐male variation in sperm length has been found for inbred males in an experimental study of *Tribolium castaneum* beetles (Michalczyk et al. 2010). In their study, however, it was unclear whether only morphologically normal sperm cells were measured.

It is likely that inbreeding severely affects male reproductive performance, particularly in species where sexual promiscuity is common and sperm competition intense (Fritzsche et al. 2006; Zajitschek et al. 2009). In birds, wide interspecific variation in levels of sexual promiscuity has been documented (Griffith et al. 2002; Lifjeld et al. 2010). If our results can be generalized to other bird species, it would suggest that inbreeding has the potential to affect ejaculate quality, and hence within‐pair paternity loss and extra‐pair paternity gain. The extent to which inbreeding depression (resulting in reduced sperm quality) actually affects male reproductive performance in avian species displaying low promiscuity, such as zebra finches (Griffith et al. 2002), remains open to further research. Reduced gamete quality, however, may compromise male fertilization success even in the absence of sperm competition (Parker 1982), and hence should be of particular concern for those involved in the management and conservation of small endangered populations (either free‐living or in captivity) that are prone to inbreeding (Gomendio et al. 2000; Wright et al. 2008).

## Ethics statement

All procedures conform to legal requirements (Permit no. 311.4‐si).

## Conflict of Interest

None declared.

## Data accessibility

Data can be accessed at Dryad (http://dx.doi.org/10.5061/dryad.4h245).
